# Big city *Bombus*: using natural history and land-use history to find significant environmental drivers in bumble-bee declines in urban development

**DOI:** 10.1098/rsos.170156

**Published:** 2017-05-17

**Authors:** Paul Glaum, Maria-Carolina Simao, Chatura Vaidya, Gordon Fitch, Benjamin Iulinao

**Affiliations:** 1 Department of Ecology and Evolutionary Biology, Ann Arbor, MI 48109, USA; 2 School of Natural Resources and Environment, University of Michigan, Ann Arbor, MI 48109, USA

**Keywords:** urbanization, pollinator, geographical information system, shrinking city, *Bombus*

## Abstract

Native bee populations are critical sources of pollination. Unfortunately, native bees are declining in abundance and diversity. Much of this decline comes from human land-use change. While the effects of large-scale agriculture on native bees are relatively well understood, the effects of urban development are less clear. Understanding urbanity's effect on native bees requires consideration of specific characteristics of both particular bee species and their urban landscape. We surveyed bumble-bee (*Bombus* spp.) abundance and diversity in gardens across multiple urban centres in southeastern Michigan. There are significant declines in *Bombus* abundance and diversity associated with urban development when measured on scales in-line with *Bombus* flight ability. These declines are entirely driven by declines in females; males showed no response to urbanization. We hypothesize that this is owing to differing foraging strategies between the sexes, and it suggests reduced *Bombus* colony density in more urban areas. While urbanity reduced *Bombus* prevalence, results in Detroit imply that ‘shrinking cities’ potentially offer unique urban paradigms that must be considered when studying wild bee ecology. Results show previously unidentified differences in the effects of urbanity on female and male bumble-bee populations and suggest that urban landscapes can be managed to support native bee conservation.

## 1. Introduction

Evidence is mounting that native wild bee populations have dramatically decreased [1–3]. The decline of these pollinators is a significant concern for human food systems, given that pollinators are responsible for the increased quantity, quality and yield stability of over 60% of world crops [4], worth an estimated approximately 200 billion dollars per year [5]. While managed honeybees have received much of the attention (especially in popular media), wild bees have also experienced significant declines. The loss of wild bee pollination is a critical concern. Wild bees are often more efficient pollinators than honeybees, providing pollination services that cannot be replaced by honeybees [6]. Furthermore, parallel declines in honeybees through colony collapse disorder reinforce the importance of wild bees to pollination services. Outside of human food systems, wild bees are essential for the maintenance of angiosperm diversity. Extirpation of bee species vulnerable to land-use change has been shown to disrupt wild plant–pollinator networks [1,7].

Causes for the decline in the abundance and diversity of wild bees are varied, though many of the empirically supported drivers have an anthropogenic source. Human land-use change has greatly altered the environment these wild pollinators inhabit through agricultural and urban development. Industrial agriculture reduces flowering plant biodiversity and suitable nesting sites, particularly for ground nesting species [8,9]. Additionally, pesticides such as neonicotinoids have been increasingly linked to declines in colony health for eusocial bees [10–13]. Neonicotinoids have also been linked to the declines seen in other wild bee species with varying degrees of sociality [14]. For urban settings, often considered detrimental for various taxa [15], the effect on wild bee communities has actually been less clear [16].

A number of published studies show no significant effects of urban development on overall wild bee abundance, richness and/or diversity [17–20]. There are studies which have found a significant negative effect of urban development on bee abundance and richness [21], though others have found this negative effect to be significant only with small solitary bees but not larger bees [22]. Still others have found that abundance declines with the highest intensities of urban development, but intermediate levels of urban development support the highest levels of species richness [23]. Finally, there are researchers who similarly found no significant effects on wild bees until sampled bees were broken down into functional groups. For example, urban environments can have very different effects on ground and cavity nesting species [24,25]. Thus, there seems to be no clear trend in the effects of urban development on overall wild bee abundance and diversity.

This lack of understanding needs to be addressed for multiple reasons. Pollinator decline is an urgent issue and global urban land area in the year 2030 is expected to be triple that of year 2000 measurements [26], meaning more and more pollinating species will come into contact with urban landscapes. Furthermore, the makeup of urban spaces is becoming more diverse. While many cities continue to expand, other cities experiencing economic hardship, deemed ‘shrinking cities’, have developed high numbers of vacant lots creating pockets of unmanaged land in supposedly dense urban locations [27]. Additionally, many modern land-use strategies now advocate the expansion of forest fragments, natural reserves and urban gardens within cities, in part, to function as potential refugia supporting biodiversity [28–31]. In other words, not only are urban spaces expanding, they are becoming more diverse while changing their form and function.

Here, we propose two issues that may be restricting the research in addressing the status of wild bees in urban environments. First, wild bees as a ‘group’ have a diverse set of natural history traits and different species probably respond differently to the same variables. For example, key differentiating traits in wild bees include nesting substrate, diet preferences and effects of sociality on bee behaviour. Studies focused on investigating specific bee species or functional groups may better elucidate how different bees respond to urban spaces. Second, urbanity is an approximate term and there is heterogeneity in what urbanization means in different cities. While measures of general physical urban development are well established [32], they may need to be coupled with further knowledge of land-use history and socio-economic characteristics of the landscape itself to develop a deeper understanding of the environment. For example, unique economic histories and different management of similar land types may alter the suitability of seemingly similar urban environments to wild bees.

To address these issues, we present an investigation on the effects of urban development on bees in the genus *Bombus* (bumble-bees) sampled across multiple cities in southeastern Michigan with varying degrees of urban development. Bumble-bees are important generalist pollinators considered a key-stone species [33,34] and are some of the most effective native pollinators [35]. Currently, numerous *Bombus* species are experiencing population and diversity declines [3,36]. Therefore, there is a conservation aim to studying *Bombus*, but bumble-bees are also suitable study organisms, given the aims of this study. The genus *Bombus* represents a distinct, well-studied set of traits that make it feasible to incorporate natural history into analysis, addressing the need to integrate species-specific traits into analysis. For example, bumble-bees' need to nest in less-disturbed areas with bare ground, tall grass or abandoned tree stumps, making them a good candidate for testing the effects of urban land development. Also, their generality as pollinators suggests less confounding effects from specific floral resources when studying *Bombus* populations across an urban gradient. Finally, the eusociality of *Bombus* means different colony members have distinct roles, behavioural and movement patterns which allows for further inference into the effects of urbanization on specific components of bumble-bee dynamics. Specifically, female workers are central place foragers, generally tied to colony location. Male drones, on the other hand, are not tied to colony location as they leave to find mates. Our study design also addresses the need to incorporate urban heterogeneity. The use of multiple city centres allows for the comparison of areas with similar general characteristics but disparate land-use histories. We contend that incorporating fundamental but potentially overlooked natural history characteristics of *Bombus* coupled with land-use history of the study sites helps present a clearer picture of the status of these bees in urban spaces.

The broad questions addressed here are:

(i) how do landscape-level variables (urbanization) and local variables (temperature, floral resources) affect measured *Bombus* abundance and diversity in sample sites?
(ii) how do the effects of urbanization differ for female workers and male drones? and
(iii) are the effects of urbanization on *Bombus* consistent in all sites across all cities sampled?

## 2. Study system

### 2.1. Sample sites

Sampling took place across 30 sites in southeastern Michigan, USA, across a gradient of urbanization during the summers of 2014 and 2015. Sites were located in the cities of Dexter, Ann Arbor, Ypsilanti, Dearborn and Detroit and span 110 km (see the electronic supplementary material, figure S1). These cities vary markedly in size and density. Detroit is a large city, but in many areas has a high proportion of vacant land, the result of decades of economic difficulty and population declines. As such—and recent economic growth notwithstanding—it, along with many other post-industrial cities experiencing population decline, has been termed a ‘shrinking city’ [27,37]. Other cities in the survey area are smaller, with substantially lower vacancy rates and with dense urban cores surrounded by suburban development. Across the 30 sites, three natural/reserve sites and two rural farms were included, while the remaining 25 were urban gardens/farms. Gardens/farms sampled in each city were either part of an independent managing organization or property of the University of Michigan (see the electronic supplementary material, table S1). Urban farms and gardens are good study sites because they act as resource lures and can have very different local characteristics. This makes it possible to study the effects of landscape-level variables by using gardens in distinctly widespread locations as well as any interactions between those landscape-level variables and different local variables at each particular garden. All sample sites prohibited the use of neonicotinoid pesticides. Garden sites have guidelines to use organic growing practices with some management organizations following the guidelines put forth by the Organic Crop Improvement Association.

### 2.2. Study organism: the genus *Bombus*

Typical bumble-bees (non-parasitic) live in colonies with a eusocial structure, including a single reproductive queen, variable numbers of non-reproductive female workers and male reproductive drones. Over-wintered, mated queens emerge, typically in spring, and begin foraging, laying eggs and producing female workers. Workers then take over the task of foraging, leaving and returning to the colony multiple times per day with pollen and nectar loads for larvae (known as central place foraging) [35]. In late summer/autumn, new virgin queens and males are produced. Both leave the colony to mate. Queens may return, but males are eventually forced out of the colony permanently. The original queen, workers and males eventually die before winter and only the newly mated queen overwinters until the next season.

Bumble-bees are generalist foragers, able to pollinate and gain sustenance from numerous plant families. Their nests are smaller than honeybee nests and are made in shaded areas within old rodent holes or self-made cavities in loose soil. There are some bumble-bees that can nest above ground in thick grass or holes in tree stumps. Bumble-bees are also strong fliers [38], able to cover greater than 1 km during foraging flights, with maximum measurements reaching approximately 2 km [39].

## 3. Material and methods

### 3.1. Bee sampling and identification

Fixed effort sampling for bees across sites was completed through pan traps and active netting. Pan traps were coated with a UV light reflective paint in one of three colours: white, yellow and blue. These three colours have shown success in covering the range of attractive UV spectrum colours used by many flowering plants [40]. We used two pan traps of each colour for a total of six pan traps per site per trapping effort. This is equal to or greater than the number of pan traps used in other studies [24,25,41,42]. In sites where vegetation height was low and the ground was visible from above, pan traps were placed at ground level. In sites where vegetation covered the ground, pan traps were mounted on PVC pipes used to match the height of the vegetation line and keep them visible to flying bees. Traps were arranged in an 8 m^2^ rectangle with pan traps at the vertices and middle of the longer sides of the rectangle. In order to cover a sufficient range of the gardens with all the pan trap colours, similar coloured pan traps were placed 2 m apart.

Bumble-bees are strong fliers and can often escape pan traps [40]. Therefore, pan trapping was accompanied by monthly active netting sessions. Netting took place each month for the duration of this study between 9.00 and 12.00 and 13.00 and 14.00 at each site during clear and sunny days (wind speeds less than 4 m s^−1^). Netting was completed using nets with a 2 ft long handle, 1 ft diameter net as well as plastic bagging when bees were stopped on flowers.

Pan trapping was performed once every second week, starting mid-May and running until mid-September for a total of nine trapping dates. Netting occurred four times throughout the sampling, once in spring, twice in summer and once at the beginning of autumn in an attempt to cover the differences in community composition linked to the major seasonal changes. Owing to permissions from managing organizations, four of the six Detroit sites had to be sampled in 2015. This additional sampling was completed in order to increase the amount of data from sites with higher urbanity. All other sites were sampled in 2014. No sites were sampled in both years. Site lists and sampling times are available in the electronic supplementary material, table S1. In general, insects are not federally regulated wildlife and no permits or permissions are required for sampling. The rusty patch bumble-bee (*Bombus affinis*) was added to the endangered species list in 2017, but this sampling took place years earlier and no *B. affinis* were collected in this dataset. Permissions for entry and sampling in sites were granted by managing organizations at each site (electronic supplementary material, table S1). All sampled bees were returned to the laboratory and stored in 70% ethanol until they were cleaned, air-dried and pinned for identification. Initial identification to sex and species was completed using a digital microscope and the discoverlife.org online key for the genus *Bombus*. Identifications were verified by taxonomist Jason Gibbs at Michigan State University.

### 3.2. Geographical information system measurements of landscape variables: impervious surface

We used geographical information system (GIS) programmes to develop profiles of the land cover types surrounding each study site. The proxy metric for urban development in this study is impervious surfaces. Impervious surfaces are roads, buildings, parking structures or anything else that effectively blankets the surface with concrete or building material. To calculate the amount of impervious surface coverage around each site, National Land Cover Database data from 2011 (Multi-Resolution Land Characteristics Consortium, mrlc.gov) was used. In keeping with McKinney's [32] suggestion of defining urban landscapes as areas with more than 50% impervious surface, areas categorized as high (80–100% impervious) and medium (50–79% impervious) density development were summed to obtain the total area of impervious surface within buffer zones of radius 500 m, 1, 1.5 and 2 km around each individual sampling site. Dividing that total area of impervious surface by the overall land area resulted in the proportional area of impervious surface cover for each buffer zone of each sampling site.

### 3.3. Local variables (floral resources and temperature)

Floral resources were measured in a 20 m radius circle at each sampling date. The circle was centred in the centre of the pan trapping 8 m^2^ rectangle on trapping dates and the centre of the netting area on active netting dates giving 1256.637 m^2^ of floral survey area per site per trapping date. Floral abundance of each species was estimated using a modified logarithmic scale (i.e. 1–10 blooms, 11–50, 51–100, 101–200, 201–500, 501–1000, greater than 1000) and species' individual floral area was calculated by averaging a representative sampling of individual flower areas for each species [43,44]. The floral area of a single species at a site can then be calculated by multiplying the flower count by the average floral area for that species; flower area has been shown to be a good proxy for floral resource availability [45]. Summing each species' area gives the overall floral resource area at each site per sampling date. Floral resource area per sample site was measured as total cumulative area across the growing season, mean area across sampling times and variance in area across sampling times. Total cumulative floral area was used as a proxy for count data to determine floral diversity per site using the Shannon–Wiener *H* index. Regressions presented here use the mean floral area per site as the site-level floral abundance, but no floral variables showed any significant effects on *Bombus* abundance or diversity in any models.

Local temperature was measured by Hobo brand data loggers from the Onset Computing Corporation placed in an unshaded area at each site within the floral survey circle. Loggers were placed at sites during the first sampling effort, removed at the last sampling date. Daily average, minimum and maximum temperatures were logged every 24 h. Several data loggers were either damaged by wildlife or stolen from sites, so temperature data were only available for 22 sites (electronic supplementary material, table S1). Temperature data across the field season were broken into three summarized subcomponents, average daily minimum temperature, average daily mean temperature and average daily highest temperature.

### 3.4. Statistical analysis

*Bombus* abundance per site is a cumulative sum of all individual bumble-bees sampled at a site. Males and females are summed separately when the abundance of different sexes are analysed. *Bombus* diversity per site was measured by using EstimateS [46] to estimate the Shannon–Wiener *H* diversity index from rarefied *Bombus* species counts. When separate female and male diversity levels are considered per site, rarefaction and Shannon–Wiener *H* estimates are completed for each sex separately.

Statistical analysis and model fitting was done using the statistical language R. *Bombus* abundance and diversity function as dependent variables in regressions, with floral resources, temperature, sampling year and the proportion of impervious surface serving as predictors. Four Detroit sites were sampled in 2015, while the remaining sites were sampled in 2014. Therefore, analysis of abundance and diversity involving the Detroit sites initially used year as a random effect in linear mixed models (LMEs) fit by maximum likelihood, with proportion of impervious surface, temperature and floral area/diversity as fixed effects. Maximum likelihood was used instead of restricted maximum likelihood in order to compare across different combinations of fixed effects [47,48]. However, across all LMEs tested, year consistently had no effect, partially because there is very little variation in the abundance and diversity of 2015 sites. Likelihood ratio tests on LMEs and general linear models (LMs) with no year effect show no significant differences. Additionally, Akaike information criterion (AIC) values for general LMs with only fixed effects are consistently lower. Therefore, the analysis involving Detroit sites presented here shows results from LM regressions. All sites outside of Detroit were sampled in 2014, so there is no year effect. Therefore, general LMs were used when analysing abundance and diversity at sites outside of Detroit alone. Model residuals show a good match to linear regression (electronic supplementary material, figures S3 and S5).

Spatial autocorrelation can influence results of regressions through effects on dependent variables. We used the same metrics as Pardee & Philpott [20] to examine the possibility of the influence of spatial autocorrelation: spatial correlograms (R package ‘ncf’) and the Moran's test for spatial autocorrelation in R. For correlograms, we computed 100 permutations using the ‘resamp’ argument in the correlog function. Moran's *I* results showed no spatial autocorrelation among dependent variables and spatial correlograms showed no spatial autocorrelation at the various buffer zone increments.

## 4. Results

Across the sample sites, we collected 520 individual *Bombus* specimens with the vast majority of the samples collected by netting (401 individuals) and a smaller subset (119 individuals) coming from pan trapping. In our sample population, 10 species/morphospecies were identified. The most abundant species sampled was *Bombus impatiens* (the common eastern bumble-bee), making up 72.12% of the sample set. Other species making up a sizeable percentage of the sample set were *Bombus griseocollis* (brown-belted bumble-bee, 11.35%) and *Bombus bimaculatus* (two-spotted bumble-bee, 9.62%), while the remaining specimens rounded out the remaining approximately 7%.

### 4.1. *Bombus* abundance

**Figure 1.**
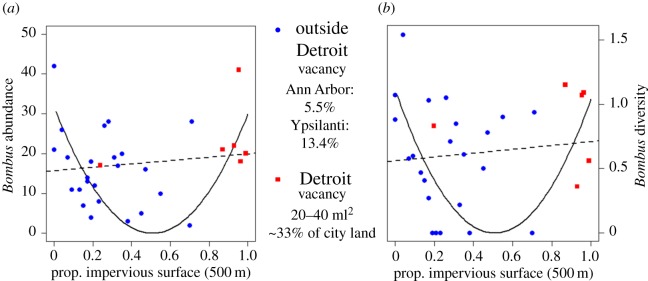
Scatterplots of (*a*) overall *Bombus* abundance and (*b*) overall *Bombus* diversity with impervious surface measured in the 500 m radii from sites. Sites outside the city limits of Detroit are shown in blue circles and sites within the city limits of Detroit are shown in red squares. (*a*) Initial linear analysis shows no significant interactions between *Bombus* abundance and % impervious space (general linear model, dashed line, *F*_1,28_ = 0.513, *p* = 0.48, *R*^2^ = −0.0171). However, a parabolic model can be significantly fitted to the data (solid line), $y={\left(a\times x-i\right)}^{2}$; *y* is the overall *Bombus* abundance, *x* the proportion of impervious surface proportion at 500 m, *a* = 11.09 with *p *< 0.001, *i* = 5.632 with *p* < 0.001. Residual standard error: 11.08 on 28 d.f. (*b*) Initial linear analysis shows no significant interactions between *Bombus* diversity and % impervious space (e.g. general linear model, dashed line, *F*_1,28_ = 0.341, *p* = 0.564, *R*^2^ = −0.0233). However, a parabolic model can be significantly fitted to the data (solid line), $y={\left(a\times x-i\right)}^{2}$; *y* is the overall *Bombus* diversity, *x* the proportion of impervious space at 500 m, *a* = 2.08 with *p* < 0.001, *i* = 1.056 with *p* < 0.001. Residual standard error: 0.474 on 28 d.f.

Initial analysis into drivers of *Bombus* abundance across all sampled sites did not indicate any significant linear relationships with impervious surface (measured at all buffer zones, figure 1*a*), floral resources (mean and total area, richness nor diversity) or temperature (low, mean nor high). However, further analysis revealed that significant parameter fits can be produced using parabolic models across proportion of impervious surface (shown at 500 m buffer zone radius in figure 1*a*). This parabolic pattern prompted investigation into the results in individual sites across the different cities along the range of impervious surface cover. Sites outside of Detroit generally aggregate on the left side of the parabola, where increasing impervious surface decreases the abundance of bumble-bees sampled. Sites within Detroit, on the other hand, are located on the right side of the parabola where increased impervious surface seemingly correlates with an increase in *Bombus* abundance compared with sites with moderate impervious surface cover.

This is an initially unintuitive trend and prompted a general examination into the characteristics of the different major urban settings of the sites, Ann Arbor, Ypsilanti and Detroit. Despite having the highest proportional area of impervious surface of any city in our study, Detroit has large amounts of vacant or idle land. Measurements of vacancy rates vary with some controversy [49], making them difficult to study/include in analysis. But recent estimates of vacancy classify approximately 33% of city land classified as vacant [50,51]. On the other hand, the other two major cities sampled, Ann Arbor and Ypsilanti, have comparatively small percentages of vacant/idle land at 8.9% and 13.4%, respectively (United States census 2010). Despite any uncertainty over the official amount of vacant land in Detroit [49], there is a clear difference in vacancy rates inside and outside Detroit. This difference in city composition (which exists despite an increase in impervious surface) signals the need for distinct analyses to be completed inside and outside of Detroit. These separate analyses serve to clarify the patterns introduced in the parabolic model fitting and help compensate for the fact that the sites with highest impervious surface were also located in the city with the highest percentage of vacant land.

**Figure 2.**
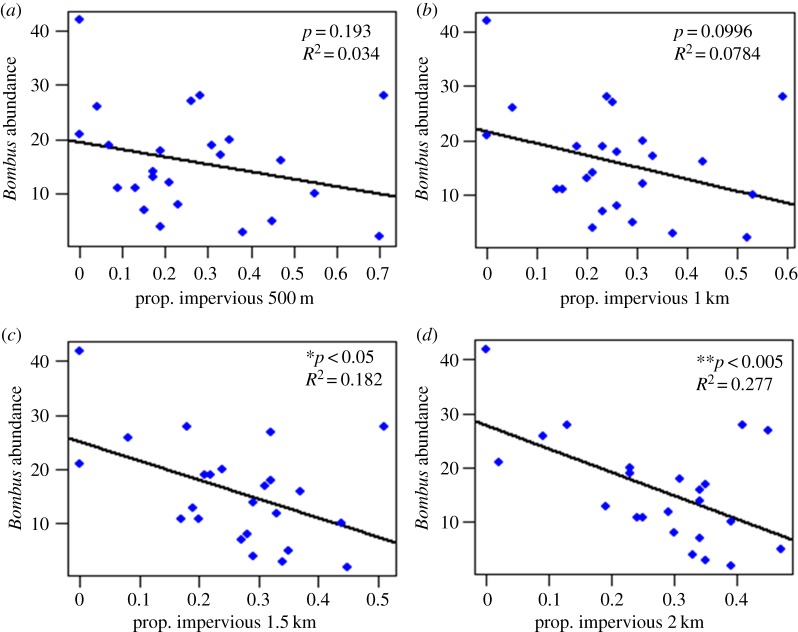
Scatterplots and general linear regressions of overall sampled *Bombus* abundance and proportion of impervious surface at sites outside of Detroit. Overall *Bombus* abundance is regressed against (*a*) 500 m buffer zone radii (*F*_1,22_ = 1.81, *p* = 0.193, *R*^2^ = 0.034, AIC = 179.97), (*b*) 1 km buffer zone radii (*F*_1,22_ = 2.96, *p* = 0.0996, *R*^2^ = 0.0784, AIC = 178.84), (*c*) 1.5 km buffer zone radii (*F*_1,22_ = 6.10, *p* = 0.022, *R*^2^ = 0.182, AIC = 176.00) and (*d*) 2 km buffer zone radii (*F*_1,22_ = 9.82, *p* = 0.0048, *R*^2^ = 0.277, AIC = 173.01). The significance of fit and effect size increase as the regressed buffer zone radius increases from 500 m to 2 km, indicating the importance of measuring landscape variables at appropriate scales. *p < 0.05; ^**^p < 0.01.

For sites outside of Detroit, general LM show significant negative correlations between impervious space and overall *Bombus* abundance. These negative correlations become stronger as the radius of the regressed environmental profile of each site increases from 500 m to 2 km (figure 2*a–d*). In other words, the effect of impervious surface on bumble-bee abundance becomes apparent only when environmental variables are measured on a larger scale. Recall that bumble-bees are strong fliers; workers have been measured flying greater than 1 km during foraging flights. If a foraging worker can fly greater than 1 km away from the colony to a particular area of floral resources, then knowing that 500 m of unsuitable habitat surrounds the floral resources does not necessarily indicate whether or not a worker will reach that resource. That is because 500 m of unsuitable space would easily be traversed by a forager with greater than 1 km of flight ability. Only upon measuring impervious surface at a scale in accordance with workers' flight ability do significant interactions become clear (electronic supplementary material, figure S2). This result highlights the importance of considering the appropriate scale when measuring environmental variables at the landscape level.

**Figure 3.**
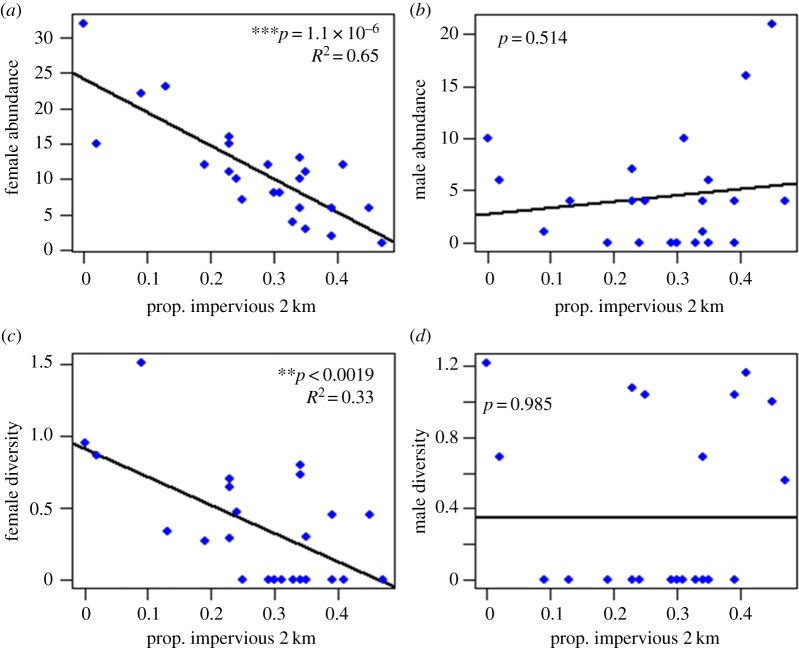
Abundance and diversity data outside Detroit split into female worker and male drone categories regressed against site-level impervious surface measured in 2 km buffer zones. Splitting data into female workers and male drones shows the decline of *Bombus* abundance and diversity in high impervious surface outside Detroit is driven by decreases in female-workers. Male drones show no significant response to impervious surface. (*a*) Female *Bombus* abundance (*F*_1,22_ = 44.08, *p* = 1.13× 10^−6^, *R*^2^ = 0.652). (*b*) Male *Bombus* abundance (*F*_1,22_ = 0.441, *p* = 0.5137, *R*^2^ = −0.0249). (*c*) Female *Bombus* diversity (*F*_1,22_ = 12.44, *p* = 0.00189, *R*^2^ = 0.3323). (*d*) Male *Bombus* diversity (*F*_1,22_ = 0.0004, *p* = 0.9852, *R*^2^ = −0.0454). ^**^p < 0.01; ^***^p < 0.001.

Owing to the apparent importance of bumble-bee flight ability, further natural history characteristics were taken into consideration. Given the behavioural differences between female workers and male drones, we separately analysed the response of each sex to impervious surface. Splitting the data reveals that the decline in overall *Bombus* abundance shown in figure 2 is entirely driven by a decrease in female workers across the impervious surface gradient (figure 3*a*). Models of reductions in female abundance follow the same pattern detailed in figure 2, becoming more significant with greater effect size as the regression considers larger buffer zone radii (electronic supplementary material, figure S4). Removing males from the regression and focusing solely on females clearly increases the significance and effect size compared with the overall abundance results outside of Detroit. On the other hand, male abundance shows no correlation with impervious surface (figure 3*b*) at any buffer radius (electronic supplementary material, figure S4). This is a strikingly different pattern between male and female bumble-bees and is consistent when examining total sampled abundance or just the most prevalent species, *B. impatiens* (electronic supplementary material, table S3).

Outside of Detroit, floral data did not seem to have an effect on *Bombus* abundance through any metric. The mean and total floral abundance per site, floral diversity and floral richness showed no significant relationships with overall *Bombus* abundance, female abundance or male abundance (electronic supplementary material, table S2). Among the sites with a temperature data logger, there is a significant negative relationship between average daily minimum temperature and *Bombus* abundance (both overall and female only, but not with male abundance, electronic supplementary material, table S4). This is owing to the link between increased impervious surface and a locations daily minimum temperature (e.g. *F*_1,19_ = 12.12, *p* = 0.0025, *R*^2^ = 0.36 w/% impervious at 500 m). In past studies, moderately higher temperatures have been linked to increased bee activity and abundance [52,53]. Here however, because minimum temperature only increases with higher amounts of impervious surface, the correlation is reversed in this study for bumble-bees.

In the Detroit sites, overall abundance showed no significant interactions or consistent trends across any of the buffer zone radii measuring impervious surface. This is partially driven by the fact that five out of six Detroit sites ended up with approximately 20 individuals sampled per site, so there is little variation in a smaller sample size. Splitting Detroit abundance data into female and male categories also shows no significant relationships. Furthermore, models including floral data do not show any significance or help with model selection. Despite the lack of correlations within Detroit sites, average abundance in Detroit sites clearly breaks from trends established with impervious surface outside of Detroit.

If abundance trends from sites outside Detroit continued in Detroit sites, models would predict close to zero bumble-bees abundance at sites with the highest impervious surface cover. However, Detroit sample sites show abundance on par with low impervious surface sites. In fact, the site with the highest impervious surface coverage (an urban agriculture demonstration garden in downtown Detroit) had only one less individual sampled than the E.S. George Nature Reserve (42 individuals), the most preserved natural site with the lowest impervious surface cover proportion in the study.

### 4.2. *Bombus* diversity

Similar to the initial patterns found in *Bombus* abundance, preliminary linear analysis on overall *Bombus* diversity shows no significant interactions or trends when considering all sites sampled in the survey. However, as with abundance, significant parameter fitting can be done using parabolic relationships between proportion of impervious surface and *Bombus* diversity (figure 1*b*). Once again, the sites outside of Detroit mainly aggregate on the left side of the parabola and sites inside Detroit largely aggregate on the right side (figure 1*b*). Given this result and the relationships found with abundance and impervious surface, separate analyses were again completed for the diversity of sites outside Detroit and sites inside Detroit.

Outside of Detroit, there are near significant declines in overall *Bombus* diversity with increased impervious surface (electronic supplementary material, table S6). Intuitively, given the different results for female and male abundance, the significant correlation between *Bombus* diversity and increased impervious surface is driven entirely by declines in female-worker diversity (figure 3*c*). The significance and effect size of impervious surface on female diversity generally increases with the radius of the environmental profile considered in the regression (electronic supplementary material, figure S6). Inclusion of floral abundance, floral diversity and temperature did not increase the goodness-of-fit for any model tested (electronic supplementary material, table S6). Male diversity, on the other hand, did not show any significant interaction with proportion of impervious surface measured at any radii (figure 3*d*; electronic supplementary material, figure S6), any floral data or temperature data.

For sites in Detroit, no significant effects of impervious surface were found for overall, female or male *Bombus* diversity. Furthermore, floral data and temperature did not aid in model fitting. Despite the lack of significant effects found across sites within Detroit, diversity of bumble-bees sampled within Detroit is higher than would be suggested by models only considering sites outside Detroit. For example, the mean diversity of females in Detroit sites is 0.52 while the data from sites outside of Detroit produce models which predict the diversity of 0.0 at approximately 70% impervious surface (electronic supplementary material, figure S6*a*). Within Detroit sites, both *Bombus* abundance and diversity go against significant relationships established by the sampling results in the remaining sites.

Finally, in sites outside of Detroit, the abundance of bumble-bees caught at each site strongly correlated with the diversity of the bumble-bees caught (*F*_1,22_ = 18.3, *p* = 0.0003, *R*^2^ = 0.43). Sites within Detroit show no such relationship (*F*_1,4_ = 0.272, *p* = 0.630, *R*^2^ = −0.171). This is partially caused by a smaller sample size within Detroit and the lack of variation in abundance found in Detroit sites.

## 5. Discussion

We found that increased urbanization (as measured through proportion of impervious surface area) in sample sites outside of Detroit had a significant negative effect on *Bombus* abundance and diversity. However, the decline is apparent only when impervious surface is measured at appropriate scales (figure 2). Bumble-bees are large-bodied bees with large foraging ranges, so measurements of landscape-level variables must be taken at scales which align with their flight ability. In fact, this study doubled the amount of land area taken into consideration of other *Bombus* studies [54]. This relationship could be lost in our efforts if impervious surface was only measured at the 500 m scale.

Crucially, we found that the decline in overall *Bombus* abundance and diversity was entirely driven by declines in female workers while male abundance and diversity were unrelated to urbanization. Given that workers are central place foragers, workers spend most of their time foraging close to the nest. Thus, it is reasonable to hypothesize that worker abundance is proportional to bumble-bee colony density. Then, this decline in worker abundance and diversity implies that higher impervious surface coverage could be reducing the number of viable *Bombus* colonies by reducing the availability of nesting sites. Such a conclusion has been supported by molecular work where urban development showed significant correlations with decreased nest density [55]. Impervious surface signifies building development, concrete parking structures, asphalt roads, etc. all forms of urban development which blanket the surface of the ground with impermeable material. This would limit species that nest underground as bumble-bees cannot dig through solid concrete. It also hinders species which nest on the surface by removing necessary cover like tall grass or tree stumps.

The lack of any relationship between male abundance and diversity with urban development is also noteworthy. Male bumble-bees are not tied to their natal colony post-emergence; rather, they disperse widely in search of mates. Our findings therefore suggest that *Bombus* are able to disperse across even highly modified urban landscapes. Considering this result, it is reasonable to hypothesize that male dispersal is potentially facilitated by the presence of urban gardens like the ones in which we sampled, as well as other green spaces that interrupt the density of impervious surfaces [56]. This distinction between female and male responses to landscape development is an important consideration for studying *Bombus* in disturbed habitats.

In addition to the importance of natural history, these results also highlight the use of considering the socio-economic history of the landscapes studied in landscape ecology. Whereas, outside of Detroit, impervious surface strongly correlated with worker decline, sites within Detroit had higher *Bombus* abundance and diversity, despite their location in the densest urban landscape. It is important to note that we do not argue that impervious surface necessarily creates a parabolic relationship with bumble-bee abundance or diversity. Instead, we argue that there is a clear negative effect of increased impervious surface on bumble-bee abundance and diversity exhibited in the 24 sites sampled outside of Detroit. The sites sampled inside Detroit, however, defy this relationship and were found to have higher abundance and diversity despite the increase in impervious surface. It was the parabolic fit that prompted splitting the analysis considering the unique context of Detroit's urban spaces.

Detroit has experienced decades of economic hardship and declining human populations. Therefore, despite its high proportional impervious surface coverage, Detroit is characterized by an abundance of vacant lots. Vacancy may make lawns more suitable as they are less frequently mowed (and compacted). They are also less likely to be treated with pesticides or herbicides. Therefore, these lots can provide various flowering plants [57,58] and suitable nesting substrate [24]. Indeed, vacant lots have been shown to support bee diversity and abundance comparable to nearby green spaces [59]. In general, our results suggest that shrinking cities present unique ecological patterns and may offer avenues for research in sustainable city development.

When addressing the decline in *Bombus* workers with increases in impervious surface outside of Detroit, it is important to consider alternative hypotheses. It is possible that impervious surfaces do not necessarily restrict the number of *Bombus* colonies, but instead correlate with a decline in the health of *Bombus* colonies such that a similar number of colonies produce fewer workers per colony than colonies surrounded by less impervious surface. However, preliminary results suggest that commercial *Bombus* colonies placed in mid and high-level impervious surface areas do not produce lower numbers of workers (C. Vaidya 2016, personal communication).

Alternatively, it could be that colony growth patterns differ systematically along the impervious surface gradient. Sampling in this study ended in September, but *Bombus* species can forage into October. If colonies in midlevel urban locations outside Detroit (where we found low *Bombus* abundance) had either later emergence time or required longer times to reach peak worker abundance, we may be underestimating *Bombus* abundance at these sites. However, the decline in *Bombus* owing to impervious surface is a consistent significant interaction across the entire sampling period, so there is no signal that this result depends on the time of year the sampling occurs (electronic supplementary material, table S5). Also, if the low abundance sites did produce more workers after our sampling ended, it is reasonable to assume there would be a similar decline in males across the urban gradient, which is not the case.

Nothing in the analysis suggested that the measured floral resources contributed to the decline in sampled workers or any of the other results presented here. While flowering plants are obviously an important resource of any pollinator, the scale of floral resources measured for this study may not align with bumble-bee foraging behaviour. Bumble-bees can make an urban garden a single stop on a longer foraging flight. Given their flight ability, floral data may need to be measured at very large scales in order to find effects of floral resources on *Bombus* prevalence.

Overall, these results have important implications for conservation of native bee populations and pollination services. The impervious surface-driven decline in *Bombus* worker abundance and diversity is potentially problematic on a broader scale, given that numerous native bee species are soil nesting and may experience similar declines. Perhaps most importantly though, is the lack of relationship between impervious surface and male abundance/diversity. This implies that female and male bumble-bees use and move through urban environments differently. This variation in movement behaviours is critical to understanding abundance patterns and an important consideration for landscape bee studies in general.

Finally, our results highlight the importance of heterogeneity in urban areas. In particular, we found that Detroit supported comparatively high native bee populations despite high amounts of impervious surface. This environmental heterogeneity should be considered more explicitly in future studies of the ecological effects of urban development. This study design and analysis framework would be well suited for replication in further *Bombus* studies in other shrinking and non-shrinking cities. A catalogue of *Bombus* response to urban development across different ecosystems and socio-economic land-use histories could potentially benefit sustainable city planning practices and would address the call for monitoring programmes for these important pollinators [60].

## Supplementary Material

General Supplementary Information

## Supplementary Material

Impervious Surface Data

## Supplementary Material

Bumble bee collections data

## Data accessibility

All pertinent data are presented in the electronic supplementary material.
