# Supplementary material for: Big city Bombus: using natural history and land-use history to find significant environmental drivers in bumble-bee declines in urban development
Source: R Soc Open Sci. 2017 May 17;4(5):170156. doi: 10.1098/rsos.170156 (PMC5451824; doi:10.1098/rsos.170156)
Supplement: General Supplementary Information [file rsos170156supp1.docx]

Supplementary Information

*Supplementary Info 1: Site Descriptions*

Table S1: Table listing sample site names, shorthand initials, location by city group, and managing organization. All sites listed were sampled in 2014 except for ACG, BM, BS, and FF which were sampled in 2015. The remaining sites could not be resampled in 2015 due to separate studies being run with bumble bees and management turnover at the sites.

| **Site Name** | **Initials** | **City Group** | **Managing Organization** | **Temperature Data Logger** |
| --- | --- | --- | --- | --- |
| Nichols Arboretum | A | Ann Arbor | U of Michigan | Yes |
| Boehnke Household | BH | Ann Arbor | Independent | Yes |
| Buhr Park | B | Ann Arbor | Project Grow | No |
| Cultivating Community | CC | Ann Arbor | U of Michigan | Yes |
| Clague Elementary | CE | Ann Arbor | Project Grow | Yes |
| Campus Farm | CF | Ann Arbor | U of Michigan | Yes |
| County Farm Park | CFP | Ann Arbor | Project Grow | Yes |
| Ellsworth | E | Ann Arbor | Project Grow | Yes |
| Greenview | GV | Ann Arbor | Project Grow | Yes |
| Leslie Science Center | LSC | Ann Arbor | Project Grow | Yes |
| Platt | P | Ann Arbor | Project Grow | Yes |
| Scio Church | SC | Ann Arbor | Independent | Yes |
| School of Public Health Garden | SPH | Ann Arbor | U of Michigan | Yes |
| West Park | WP | Ann Arbor | Project Grow | Yes |
| UM-Old Field | OF | Dearborn | U of M-Dearborn | No |
| UM-Organic Garden | OG | Dearborn | U of M-Dearborn | Yes |
| Art Center Community Garden | ACG | Detroit | Midtown Gardens | No |
| Brightmoor Foodway | BM | Detroit | Independent | No |
| Burnside Community Garden | BS | Detroit | Independent | No |
| Food Field | FF | Detroit | Independent | No |
| Lafayette Greens | LG | Detroit | Greening of Detroit | Yes |
| N. Cass Community Garden | NC | Detroit | Midtown Gardens | No |
| Dexter Community Garden | DCG | Dexter | Independent | Yes |
| E.S. George Reserve | ESG | Dexter | U of Michigan | Yes |
| M'Lis Farm | MF | Dexter | Independent | Yes |
| Catholic Social Services | CSS | Ypsilanti | Growing Hope | Yes |
| EMU - The Giving Garden | EMU | Ypsilanti | Growing Hope | Yes |
| Frog Island Community Garden | FI | Ypsilanti | Growing Hope | Yes |
| Normal Park Community Garden | NP | Ypsilanti | Growing Hope | Yes |
| Perry / Parkridge Community Garden | PCG | Ypsilanti | Growing Hope | No |


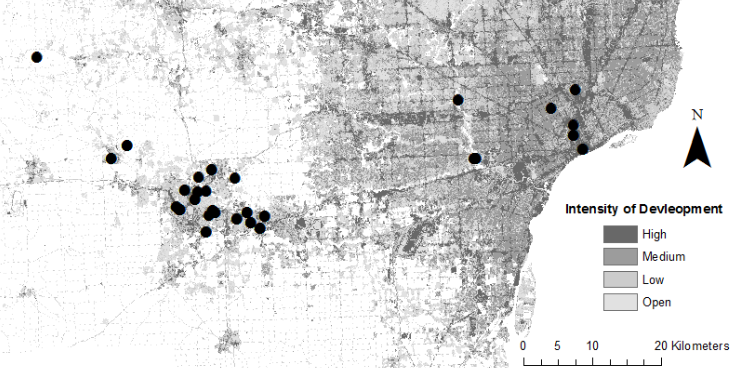


Figure S1: GIS map of southeastern Michigan. Black dots represent sites where sampling occurred. The proportion of impervious surface coverage (Intensity of Development) at a given location (see Methods) is given by the grayscale gradient. Landscape data sourced from 2011 National Landcover Database Multi-Resolution Land Characteristics Consortium (MRLC) mfc.gov.


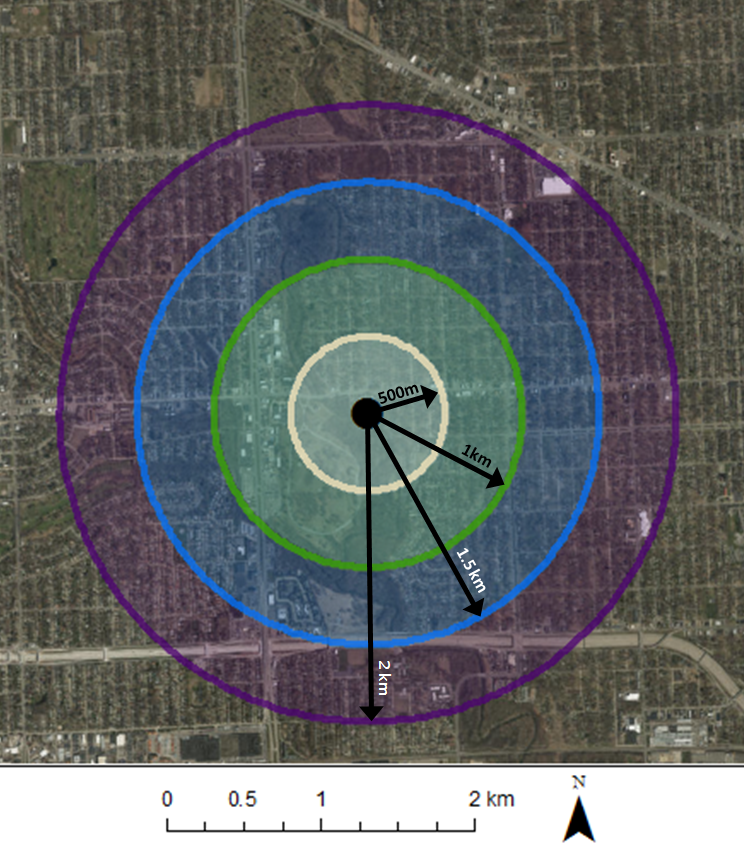


Figure S2: Figure representing the four buffer zones inside of which the proportion of impervious surface was measured around each site. The four buffer zones had distinct radii of 500m (white circle), 1km (green circle), 1.5km (blue circle), and 2km (purple circle). The significance of the regressions shown in Figures 2 (in the main paper), Fig S4, and Fig S6 increased as the buffer zone radius increased. For example, in Figure 2, when impervious surface coverage was measured within 2km of each site, the regression produced most significant model output with the highest effect size. Measuring impervious surface at only 500m provided an insufficient measurement of impervious surface and consequently the signal was lost.

*Supplementary Info 2: Abundance*

Table S2: Summary stats of the effects of all measured independent variables on female *Bombus* abundance outside of Detroit from general linear models. All sites outside of Detroit were measured in 2014 so use of a mixed model is not required. The linear effect of Proportion of Impervious 2km is clearly the best fit model.


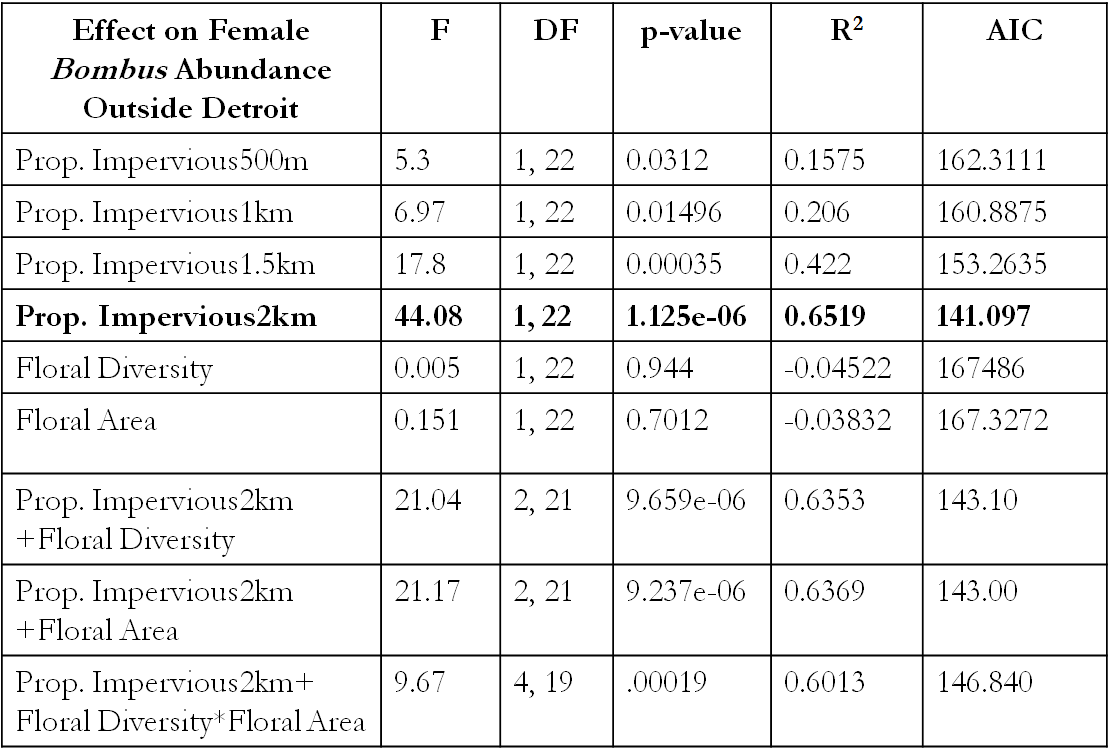


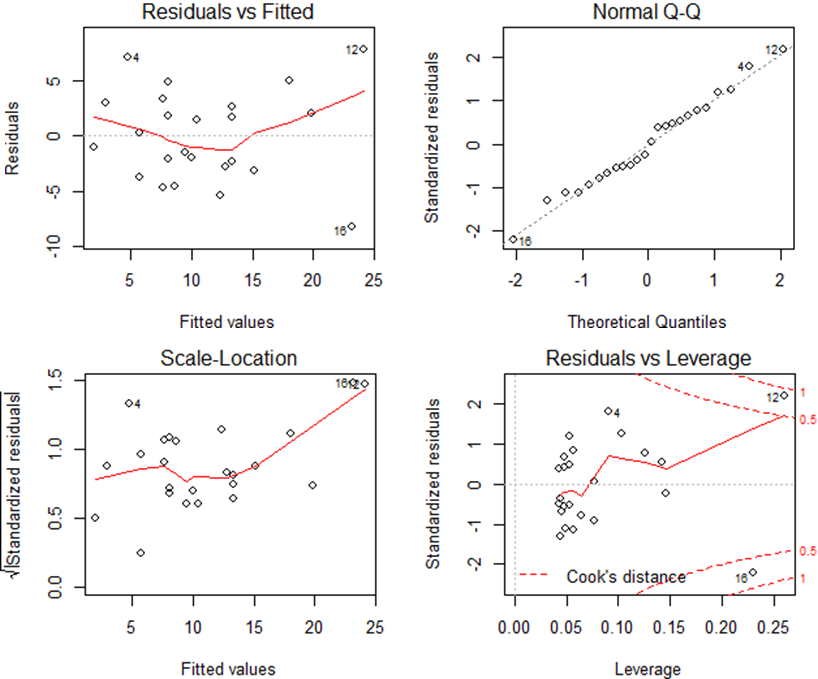


Figure S3: Summary plots for the linear model female *Bombus* abundance ~ %Impervious 2km from Table S2. Only sites outside of Detroit considered here.

Table S3: The different effect of impervious surface on the overall females and males communities is also apparent when looking only at the most abundant species (*B. impatiens*). There is a strong negative relationship for females and no significant patterns for males. Only sites outside of Detroit considered here.


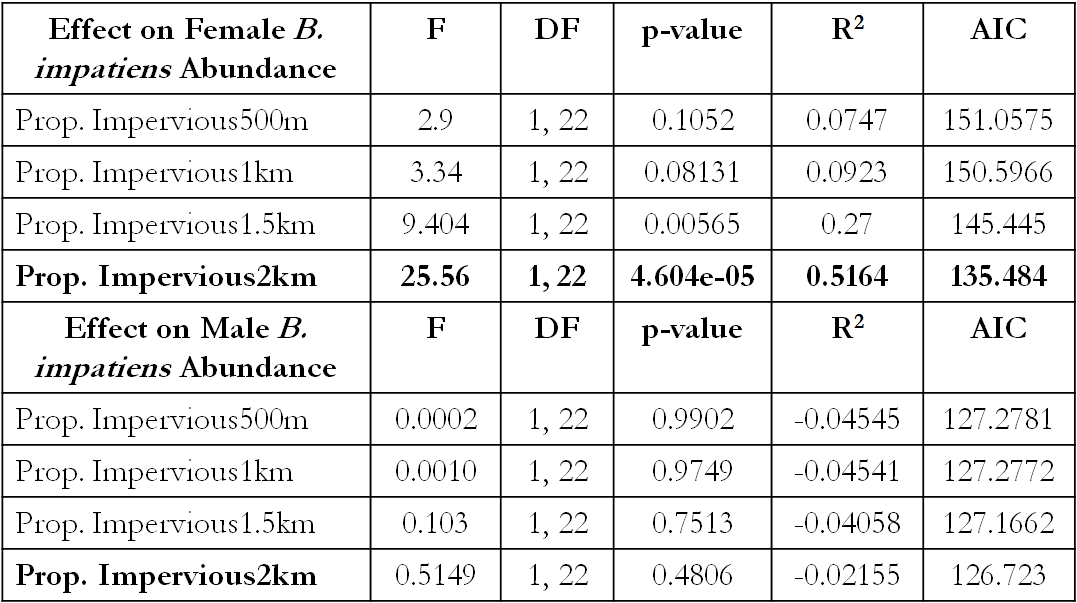


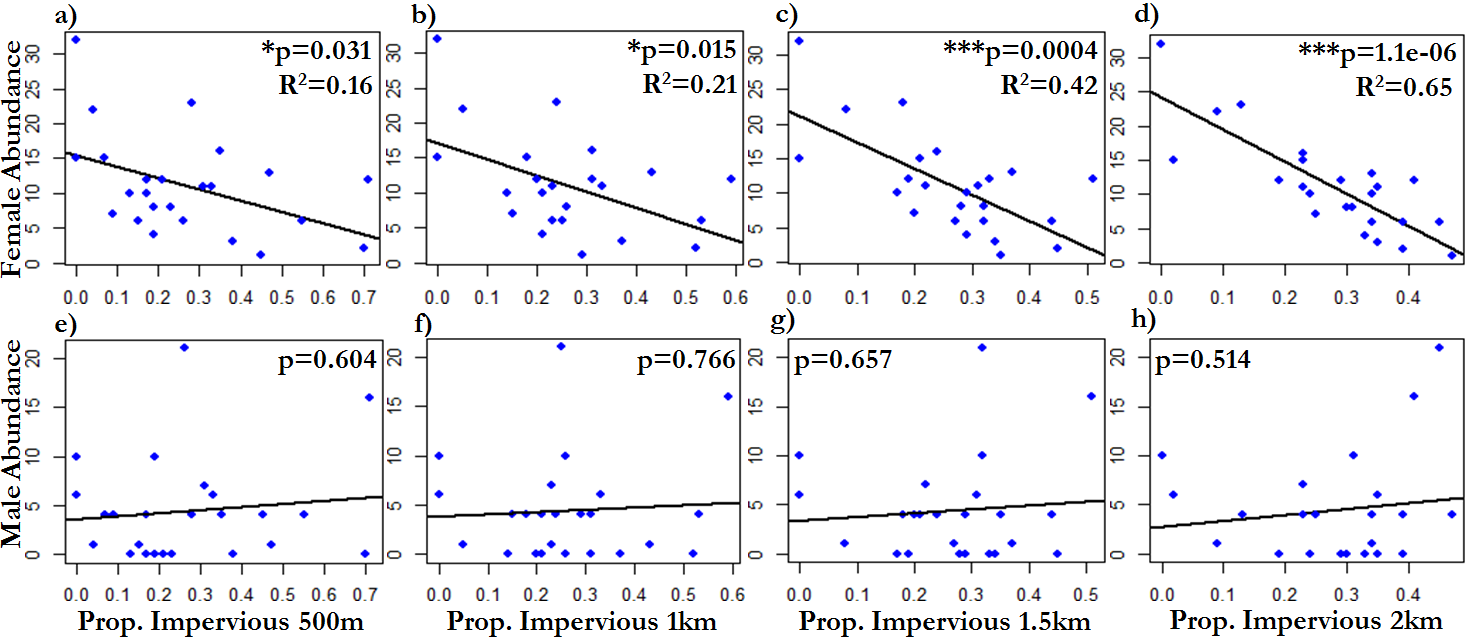


Figure S4: Abundance data outside of Detroit split into female worker and male drone categories regressed across the impervious surface gradient with general linear models. Splitting abundance data into female workers and male drones shows the decline of *Bombus* abundance in high impervious surface is driven by decreases in female-worker abundance outside of Detroit. Female abundance regressed against a) 500m (F_1,22_ = 5.3, p= 0.0312, R^2^ = 0.158), b) 1km (F_1,22_ = 6.97, p= 0.01496, R^2^ = 0.206), c) 1.5km (F_1,22_ = 17.8, p= 0.00035, R^2^ = 0.422), d) 2km (F_1,22_ = 44.08, p= 1.125e^-6^, R^2^ = 0.652). Male abundance regressed against e) 500m (F_1,22_ = 0.28, p= 0.604, R^2^ = -0.0325), f) 1km (F_1,22_ = 0.091, p= 0.766, R^2^ = -0.0412), g) 1.5km (F_1,22_ = 0.202, p= 0.657, R^2^ = -0.0359), h) 2km (F_1,22_ = 0.441, p= 0.514, R^2^ =-0.0249).

*Supplementary Info 3: Temperature*

Increases in impervious surface are highly correlated with an increase in the lowest daily temperature measured at sites. Urban centers are heat islands which tend to trap heat from day time temperatures. This causes locations with higher amounts of impervious surface to have higher minimum temperatures across the span of a day. Without impervious surfaces, heat is not trapped in concrete structures and is released, therefore leading to a lower minimum daily temperature.

Table S4: Model output from sites where temperature was recorded with a data logger. The best model including the temperature data is still %Impervious surface at 2km for *Bombus* abundance.


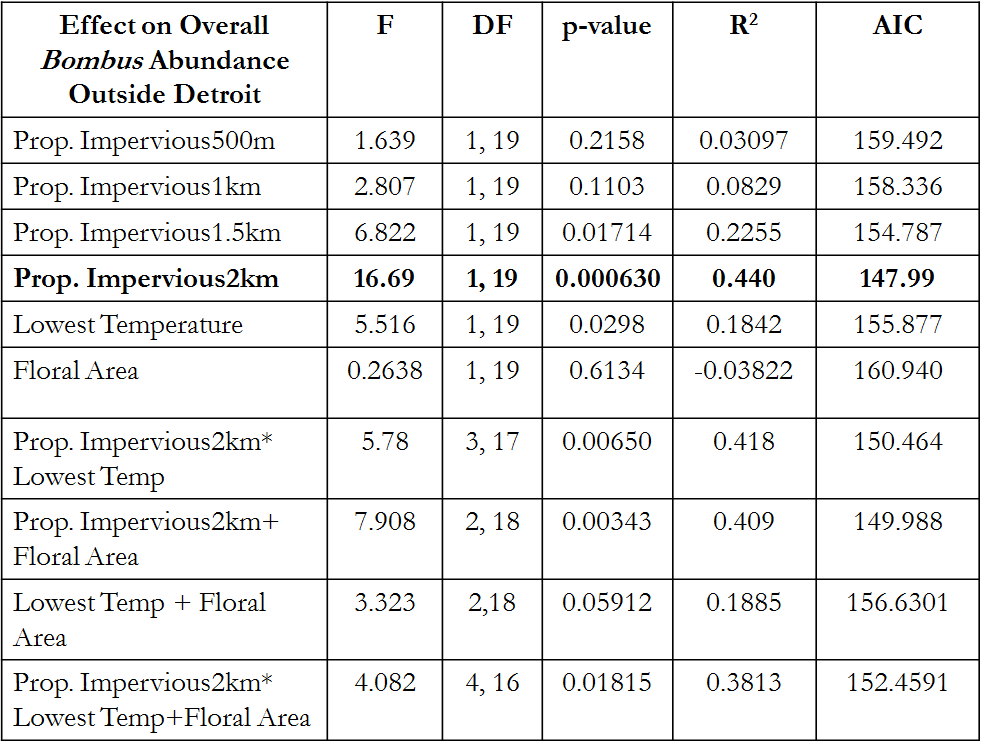


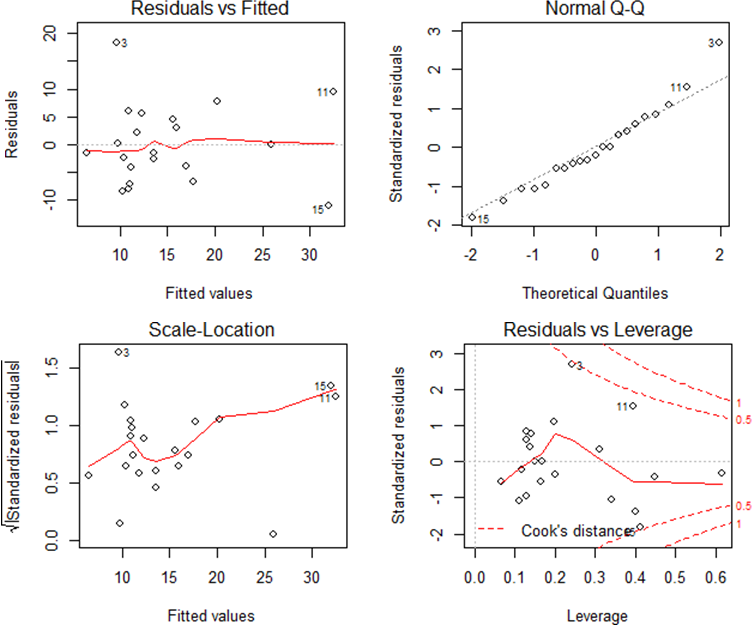


Figure S5: Summary plots for the model *Bombus* abundance ~ lowest temp * %Impervious 2km + floral area from Table S4. Only sites outside of Detroit considered here.

*Supplementary Info 4: Addressing alternative hypotheses*

In addressing alternate hypotheses for the decline in worker abundance and diversity outside of Detroit, we consider the possibility that the sampling schedule of this study missed a potential delayed emergence or growth rate of *Bombus* in mid-level impervious surface coverage. If this was the case, then it is likely that the measured decrease in *Bombus* with higher impervious surfaces would be limited to earlier times of the year and become insignificant later in the year. To test this, the overall *Bombus* sampling data was split into four distinct periods across the field season to align with the four netting dates instead of the cumulative sum over the entire growing as was used in the main analysis. This was done because the vast majority of samples came from netting efforts. Each period then also included the samples from the trapping prior to the netting date and the trapping immediately after the netting date. This then allowed us to test the consistency of the impervious surface induced worker decline across smaller subsections of the overall field season. The four periods are titled P1, P2, P3, and P4.

Worker decline with impervious space is significant in P1 and P3 and the decline is very nearly significant in P2 (Table S5). The decline initially seemed to disappear in P4, but this was found to be an artifact of sampling in farms post crop harvest. The two farms included in this study (MF and CF) had been harvested and cleared prior to final netting date. This removed nearly all floral resources from each site and limited sampling efficacy as workers did not land in our sampling area. While measured floral resources in this study were found to not significantly affect *Bombus* abundance, a complete absence of flowers is enough to eliminate any ability to lure and catch foraging workers. Removing those two sites from the analysis shows that the decline in *Bombus* with impervious surface is still significant in P4.

While there is certainly variation in the effect size and significance, but all periods analyzed show the same decline in relation to impervious surface and there is no consistent weakening of the trend across the summer. Therefore, the significant decline in *Bombus* workers due to impervious surface seems to be a consistent result across the entire sampling season. There is no evidence of a later increase in *Bombus* activity that disrupts the signal.

Table S5: This table shows the consistent significance of the negative effect of impervious surface on female *Bombus* abundance outside of Detroit across the growing season. Periods represent the sum total female abundance of trappings and nettings from 4 distinct periods in growing season instead of the entire growing season. Each period contains one netting date and two pan trapping dates. Impervious surface is measured in the 2km buffer.


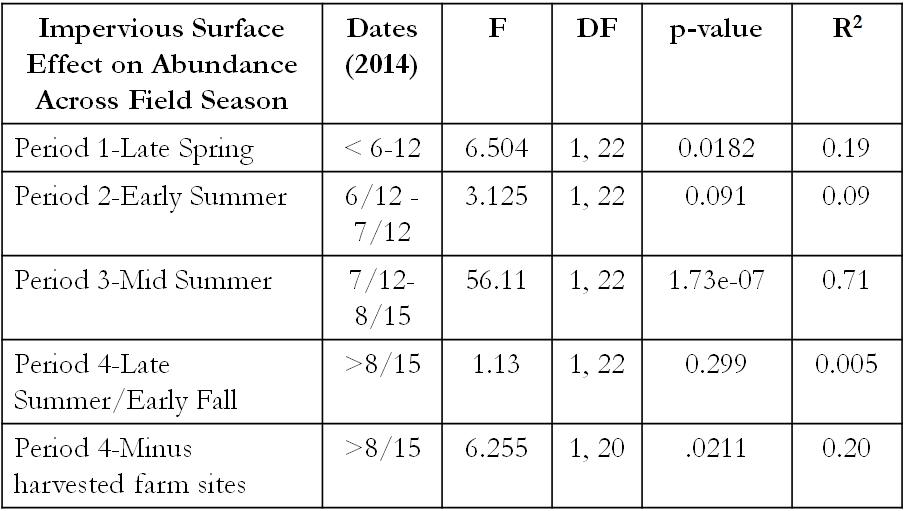


*Supplementary Info 5: Diversity*

Table S6: Summary stats of the effects of all measured independent variables on overall *Bombus* diversity outside of Detroit.


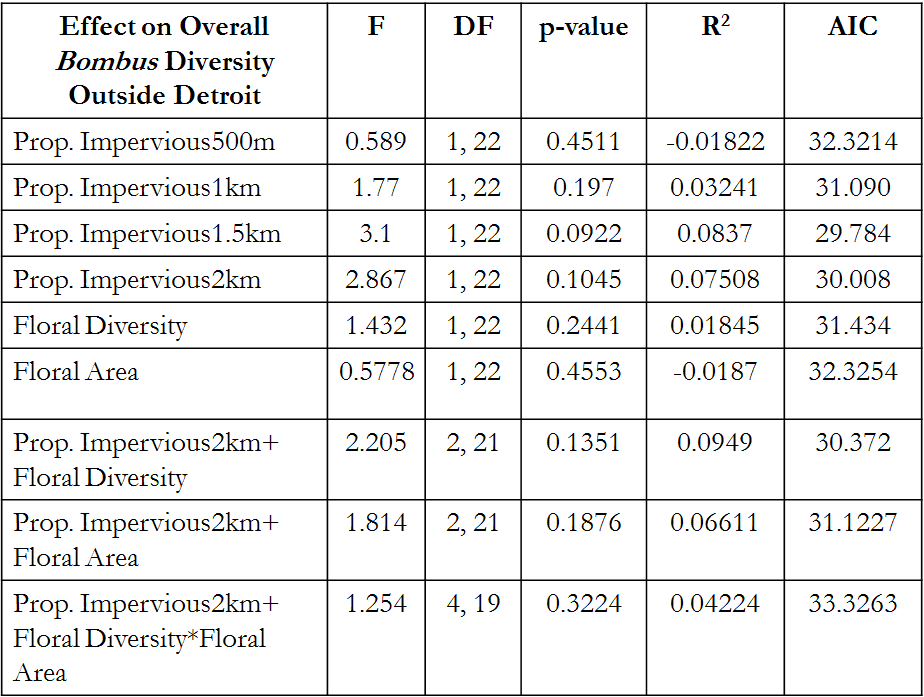


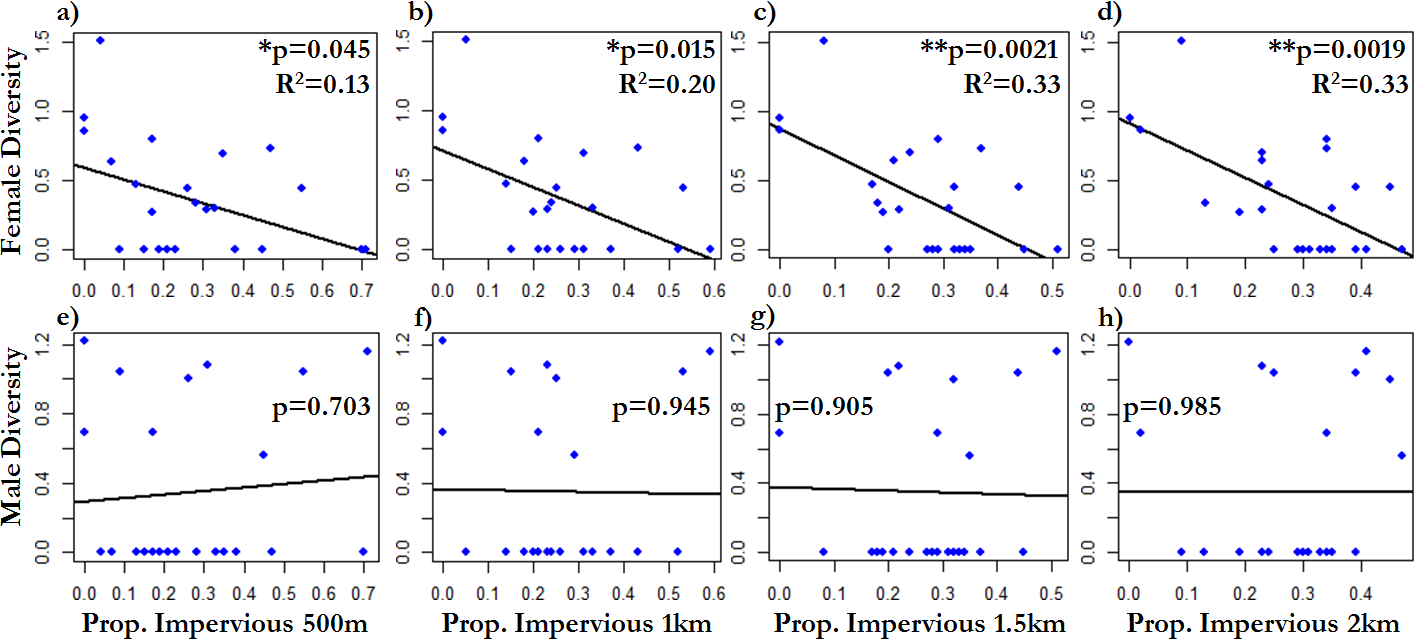


Figure S6: Diversity data outside of Detroit split into female worker and male drone categories regressed across the impervious surface gradient with general linear models. Splitting diversity data into female workers and male drones shows the decline of *Bombus* diversity in high impervious surface is driven by decreases in female-worker diversity outside of Detroit. Female diversity regressed against a) 500m (F_1,22_ = 4.53, p = 0.0448, R^2^ = 0.134), b) 1km (F_1,22_ = 6.895, p = 0.0154, R^2^ = 0.204), c) 1.5km (F_1,22_ = 12.2, p = 0.0021, R^2^ = 0.326), d) 2km (F_1,22_ = 12.44, p = 0.0019, R^2^ = 0.332). Male diversity regressed against e) 500m (F_1,22_ = 0.150, p = 0.703, R^2^ = -0.0384), f) 1km (F_1,22_ = 0.00049, p = 0.945, R^2^ = -0.0452), g) 1.5km (F_1,22_ = 0.0148, p = 0.905, R^2^ = -0.0448), h) 2km (F_1,22_ = 0.00035, p= 0.985, R^2^ =-0.0454).
